# Cytotoxic activity of non-specific lipid transfer protein (nsLTP1) from Ajwain *(Trachyspermum ammi)* seeds

**DOI:** 10.1186/s12906-022-03616-y

**Published:** 2022-05-16

**Authors:** Saud O. Alshammari, Taibah Aldakhil, Qamar A. Alshammari, David Salehi, Aftab Ahmed

**Affiliations:** 1 Biomedical and Pharmaceutical Sciences, Chapman University School of Pharmacy, 9401 Jeronimo Road, Irvine, CA 92618 USA; 2 Department of Plant Chemistry and Natural Products, Faculty of Pharmacy, Northern Border University, Arar, Saudi Arabia; 3 Department of Pharmaceutical Chemistry, College of Pharmacy, Prince Sattam Bin Abdulaziz University, Al-Kharj, Saudi Arabia; 4 Department of Pharmacology and Toxicology, Faculty of Pharmacy, Northern Border University, Arar, Saudi Arabia

**Keywords:** Ajwain seeds, Protein, nsLTP, Anticancer activity, Apoptosis, Serum stability, CD spectroscopy, Flow cytometry

## Abstract

**Background:**

*Trachyspermum ammi,* commonly known as Ajwain, is a member of the Apiaceae family. It is a therapeutic herbal spice with diverse pharmacological properties, used in traditional medicine for various ailments. However, all previous studies were conducted using small molecule extracts, leaving the protein’s bioactivity undiscovered.

**Aim:**

The current study aimed to demonstrate the cytotoxic activity of Ajwain non-specific lipid transfer protein (nsLTP1) in normal breast (MCF10A), breast cancer (MCF-7), and pancreatic cancer (AsPC-1) cell lines. Also, to evaluate its structural stability in human serum as well as at high temperature conditions.

**Methods:**

The cytotoxic activity of Ajwain nsLTP1 was evaluated in MCF-7 and AsPC-1 cell lines using MTT assay. Annexin V-FITC and PI staining were used to detect the early apoptotic and late apoptotic cells. The role of nsLTP1 in inducing apoptosis was further studied by quantifying Bcl-2, Bax, Caspase-3, Survivin, EGFR, and VEGF genes expression using RT-PCR. CD spectroscopy analyzed the nsLTP1 conformational changes after thermal treatment for structure stability determination. The RP-HPLC was used to analyze the nsLTP1 degradation rate in human serum at different time intervals incubated at 37 °C.

**Results:**

Ajwain nsLTP1 showed a potent cytotoxic effect in MCF-7 and AsPC-1. The IC_50_ value obtained in MCF-7 was 8.21 μM, while for AsPC-1 4.17 μM. The effect of nsLTP1 on stimulating apoptosis revealed that the proportions of apoptotic cells in both cell lines were relatively increased depending on the concentration. The apoptotic cells percentage at 20 μM was in MCF-7 71% (***P < 0.001) and AsPC-1 88% (***P < 0.001). These results indicate that nsLTP1 might efficaciously induce apoptosis in multiple types of cancerous cells. Genes expression in MCF-7 and AsPC-1 showed significant upregulation in Bax and Caspase-3 and downregulation in Bcl-2, Survivin, EGFR, and VEGF protein. The CD analysis of nsLTP1 showed a significant thermostable property. In serum, nsLTP1 showed a slow degradation rate, indicating high stability with a half-life of ~ 8.4 h.

**Conclusion:**

Our results revealed the potential anticancer activity of Ajwain nsLTP1 and its mechanism in inducing apoptosis. It further exhibited thermostable properties at high temperatures and in human serum, which suggested this protein as a promising anticancer agent.

**Supplementary Information:**

The online version contains supplementary material available at 10.1186/s12906-022-03616-y.

## Background

Life-threatening diseases have become one of the most global healthcare concerns [1]. However, cancer is still one of the most significant causes of death with high morbidity and mortality. According to International Agency for Research on Cancer, the globally estimated number of cancer cases in 2020 was 19.3 million. In 112 countries, cancer is considered the first leading cause of death in the young population, with almost 10 million deaths in 2020 [2]. Additionally, the United States alone has 1,806,590 new cancer cases and 606,520 cancer deaths in the last year. Among different cancer types, breast cancer is the most diagnosed cancer in women, with 2.3 million new cases in 2020 [3]. Moreover, it has been reported that breast cancer families with BRCA1 and BRCA2 mutations have been linked to an increased risk of pancreatic cancer [4]. Despite all advanced technologies and new treatment approaches, the cancer survival rate is still very low for multiple types, including the pancreas (9%) [3].

Circumvention of apoptosis is a common feature of all malignant cells. There is an imbalance between cell division and cell death in cancerous cells due to sequential genetic changes and alterations in the signal transduction pathways resulting in deregulation of the apoptosis process [5, 6]. Understanding the apoptosis process is extremely important because it gives insight into the pathogenesis of disease and the possible treatment mechanisms. Apoptosis is a programmed mechanism of cell death activated in response to cell stress or growth factor deprivation. It is initiated by either the mitochondrial (intrinsic) pathway or the death receptor (extrinsic) pathway [7]. Both paths lead to the activation of caspases, which are responsible for DNA and protein breakdown. Therefore, down-regulation of anti-apoptotic proteins and up-regulation of proapoptotic proteins will enhance anticancer activity by inducing apoptosis [8]. Current treatment approaches such as surgery, radiation, and small molecules chemotherapy have massive side effects, which demand researchers and pharmaceutical industries to develop a new effective treatment with fewer adverse effects. Protein-based drugs extracted from plants and other natural organisms might have a significant role in drug development and the discovery for new cancer agents [9–11].

Based on multiple reports, nonspecific Lipid Transfer Proteins (nsLTPs) possess anticancer activities against several cancer types. Plant nsLTPs are cysteine-rich proteins classified based on their molecular mass into two subfamilies, nsLTP1 (9–10 kDa) and nsLTP2 (6–7 kDa) [12]. Their structure is stabilized by four disulfide bonds formed between eight cysteine residues [13]. Functionally, they are characterized by their ability to bind with lipid molecules and transfer them between membranes. They are also involved in the plant’s innate immunity, especially the signaling pathways [14]. Both nsLTPs were successfully purified and characterized from various plants species [15–22]. In the in-vitro experiments, nsLTPs from fennel seeds (*Foeniculum vulgare*) and cole seeds (*Brassica campestris*) showed anticancer activity against breast cancer cell line (MCF-7) [23, 24]. In addition, the cytotoxic effect of nsLTP isolated from *Peganum harmala* seeds has been observed in four cancer cell lines, including melanoma (B16), esophagus carcinoma (Eca-109), gastric carcinoma (MGC-7), and cervical carcinoma (HeLa) [25]. Despite all discovered nsLTPs from different plants, their cytotoxic activity studies remain limited. Previously, we have reported the extraction, purification, amino acid sequence, and molecular modeling of nsLTP from Ajwain seeds [26]. The complete primary structure established is deposited to the UniProt Knowledgebase under the accession number C0HLG2, https://www.uniprot.org/uniport/C0HLG2. Subsequently, our in silico investigation presents 3D modeling, docking patterns, and dynamic interactions of Ajwain nsLTP1 with different ligands. The coordinate file of Ajwain nsLTP1 is submitted to the online protein model database (PMDB). The PMDB ID of the submitted model is PM0081852. This study evaluates the nsLTP1 cytotoxic activity extracted from Ajwain (*Trachyspermum ammi* L.) seeds. The Ajwain is also known as ajowan, caraway, bishop’s weed, or carom seeds. It belongs to the plant family Umbelliferae or Apiaceae. Members of this family are well known for their therapeutic properties. In traditional medicine, Ajwain seeds are commonly used to relieve various ailments such as colic, dyspepsia, and diarrhea [27]. Also, previous experiments showed pharmacological properties, including antibacterial [28, 29], antifungal [30], anthelmintic [31], antihypertensive, hepatoprotective [32], anti-inflammatory [33], and antioxidant [34]. However, all explored bioactive properties of Ajwain seeds were based on aqueous, acetonic, ethanolic, methanolic, and essential oil extracts, leaving the proteins and peptides undiscovered [35]. Therefore, this study is focused on evaluating nsLTP1 cytotoxic activities in breast cancer (MCF-7) and pancreatic cancer (AsPC-1) cell lines, quantified the expression of pro- and anti-apoptotic genes to investigate the ability of nsLTP1 in inducing apoptosis and its anticancer mechanism. Furthermore, the stability of nsLTP1 in human serum was evaluated in an in-vitro assay and its secondary conformations at different temperatures using circular dichroism (CD) spectroscopy.

## Materials and methods

Ajwain seeds were deposited and authenticated based on morphology by Dr. Muneeba Khan, Taxonomist at the Center for Plant Conservation, University of Karachi Herbarium and Botanical Garden. As per institutional policy, seeds are not accepted as a voucher specimen, but no deposition number is issued, required for the whole plant.

### Extraction and purification of nsLTP protein

The extraction and the purification of nsLTP from defatted Ajwain seeds have been reported previously [26]. The purified nsLTP protein was further characterized for the secondary protein structure, serum stability and evaluated in vitro cytotoxic activities in two different cancer cell lines and a normal cell line.

### Normal and cancer cell culture

Human breast cancer MCF-7 (ATCC HTB-22), pancreatic cancer AsPC-1 (ATCC CRL-1682), and normal breast MCF10A (ATCC CRL-10317) cell lines (ATCC, Manassas, USA) were cultured at 37 °C with 5% CO_2_. The MCF-7 cancer cells were maintained in Dulbecco’s Modified Eagle Medium (DMEM) and AsPC-1 in the Roswell Park Memorial Institute (RPMI) mediums, supplemented with 10% (v/v) of fetal bovine serum (FBS) and 1% (v/v) penicillin-streptomycin. The normal breast cells MCF10A was maintained in mammary epithelial cell basal medium (MEBM), supplemented with mammary epithelial cell growth medium (MEGM) growth factors (Lonza, USA).

### Cell survival assay using MTT

The cytotoxic effect was determined using purified nsLTP1 by RP-HPLC. The sample was concentrated, and the protein concentration was estimated by Bradford assay. The sample for cell culture was prepared in the respective medium and evaluated using MTT [3-(4, 5-dimethylthiazol-2-yl)-2, 5-diphenyl tetrazolium bromide] assay [36]. In 96 well plates, 200 μL of medium containing 10,000 cells/well were seeded and incubated for 24 h at 37 °C with 5% CO_2_. The medium was removed the next day, and different concentrations (2.5, 5, 10, 15, and 20 μM) of purified nsLTP1 were used to treat the cells and calculated the IC_50_ for both cell lines. The Doxorubicin was used as a positive control, whereas untreated cells were control cells in this experiment. After 48 h incubation, the treatments were replaced with MTT dye (0.5 mg/mL in medium), followed by 4 h incubation at 37 °C and 5% CO_2_. After discarding the dye, 100 μL DMSO was added to solubilize the purple-blue colored formazan crystal. SpectraMax M5 Microplate Reader was used to measure the absorbance at 550 nm. This experiment was repeated three times in triplicate, and the following equation was used to calculate the percentage of inhibition:

$$\%\mathrm{Inhibition}=\frac{\mathrm{Absorbance}\ \mathrm{of}\ \mathrm{control}-\mathrm{Absorbance}\ \mathrm{of}\ \mathrm{treated}\ \mathrm{cells}}{\mathrm{Absorbance}\ \mathrm{of}\ \mathrm{control}}\mathrm{x}100$$

### Apoptosis assay by flow cytometry

Cell apoptosis detection assay was performed using a commercially available kit (FITC Annexin V Apoptosis Detection Kit II, BD Biosciences, USA) as per the manufacturer’s instructions. We used three different concentrations to see if the degree of apoptosis is affected by changing the concentration. Briefly, 2.5 × 10^5^ cells per 2 mL were seeded in each well in a 6-well plate and were allowed to adhere to the bottom of the plate for 24 h in the incubator at 37 °C in a humidified atmosphere of 5% CO_2_. After 24 h, the cells were inspected for their health and confluency. After that, the cells were treated with nsLTP1 (5, 10, and 20 μM) for 48 h. The untreated cells served as control. After 48 h treatment, the cells were harvested and centrifuged at 1500 RPM. The pellet was washed twice using cold PBS and resuspended using the 1X binding buffer. Annexin V (5 μL) and propidium iodide (PI) (5 μL) were mixed with 100 μL of the suspended cells in a culture tube, followed by 15 min incubation at room temperature in the dark. The mixture was diluted by adding 400 μL of the 1X binding buffer and immediately analyzed using flow cytometry, FACS Verse (BD Biosciences, USA). This experiment was repeated independently three times in triplicate [37].

### RNA extraction, cDNA synthesis, and gene expression quantification

MCF-7 and AsPC-1 cells were seeded in 6-well plates at a density of 2.5 × 10^5^ cells/well for 24 h. The cells were treated with different concentrations of nsLTP1 (5, 10, and 20 μM) for 48 h. TRIzol reagent (Invitrogen, USA) was used to extract mRNA according to the manufacturer’s protocol. The RNA concentration was quantified using a NanoDrop spectrophotometer (Thermo Fisher Scientific, USA). The yields were approximately 2 μg from each cell line which were reverse transcribed to cDNA using the RevertAid First Strand cDNA synthesis kit (Thermo Fisher Scientific). The cDNA was used to quantify Bcl2, BAX, caspase-3, surviving, EGFR, and VEGF gene expressions by Real-Time Polymerase Chain Reaction (RT-PCR). Reactions were performed using Maxima SYBR Green/ROX qPCR master mix kit (Thermo Fisher Scientific) in QuantStudio 3 Real-Time thermocycler (Applied Biosystems, Thermo Fisher Scientific) under the following conditions: 95 °C for 10 min followed by 40 cycles of 95 °C for 15 s and 55 °C for 60 s. GAPDH was used as the housekeeping gene. Fold change in gene expression was calculated using the 2^-ΔΔCt^ method and presented as the mean ± SD relative to control cells in three independent triplicate experiments [38].

### Circular Dichroism (CD) spectroscopy

MilliQ water was used to make a 50 μM concentration of nsLTP1 for the CD spectroscopy analysis. MilliQ water background-subtracted CD spectra were recorded using a JASCO J-1500 spectropolarimeter equipped with the Peltier Jasco PTC-510 temperature controller (Jasco Corp., Japan) under the following conditions: Nitrogen flow 20 SCFH, PM-539 detector, data interval 0.1 nm, data pitch 0.1 nm, CD scale 200 mdeg/0.1 dOD, FL scale 200 mdeg/0.1 dOD, bandwidth 1 nm, cell length 1 mm, start mode immediately, scanning mode continuous, scanning speed 50 nm/min, and shutter control auto. An average of three readings between 190 and 260 nm was collected for each sample. The sample was scanned at 20, 40, 60, 80, and 95 °C for 20 min, and then cooled to 20 °C for 20 min before being scanned again. Under the same conditions and parameters, collagen was used as a control to compare the conformational changes in the secondary structure after thermal treatment. Analysis was carried out using one of the multivariate regression analyses, Principal Component Regression (PCR), with a basis set containing 26 proteins reference set under the following conditions: standardization of result 100, replaced negative value to zero, and rejection percentage 1%.

### In vitro stability assay in human serum and medium containing FBS

Analytical RP-HPLC was used to analyze the nsLTP1 stability studies in human serum (MP Biomedicals, USA) and medium containing FBS. Human serum 25% in water and the cell culture medium containing 10% FBS were mixed with 20 μM of nsLTP1 and incubated at 37 °C, respectively. At different time intervals (0, 3, 6, 12, 24, 48, and 72 h), several aliquots (100 μL) were diluted in 200 μL cold methanol and kept for 10 min on ice to precipitate the proteins. The samples were centrifuged at 10000 RPM for 10 min; the supernatant was injected into Aeris Widepore-C4 particle size 3.6 μm, pore size 200 Å (250 × 4.6 mm) column running with a gradient 0–60% acetonitrile with 0.1% trifluoroacetic acid (TFA) in 65 min. The elution flow rate was 1 mL/min, and the absorbance was detected at 214 nm. The degradation of nsLTP1 was confirmed by measuring the area under the curve (AUC) after each run.

### Statistical analysis

GraphPad Prism 9.3.1 software (La Jolla, CA, USA) was used to analyze the experimental data. One-way ANOVA (analysis of variance) was used for group comparison, followed by Dunnett’s post-hoc test to determine the statistical significance. Results with *P* values of < 0.05 were considered statistically significant and presented as *P < 0.05, **P < 0.01, ***P < 0.001.

## Results

### Cytotoxic effect of nsLTP1

**Fig. 1:**
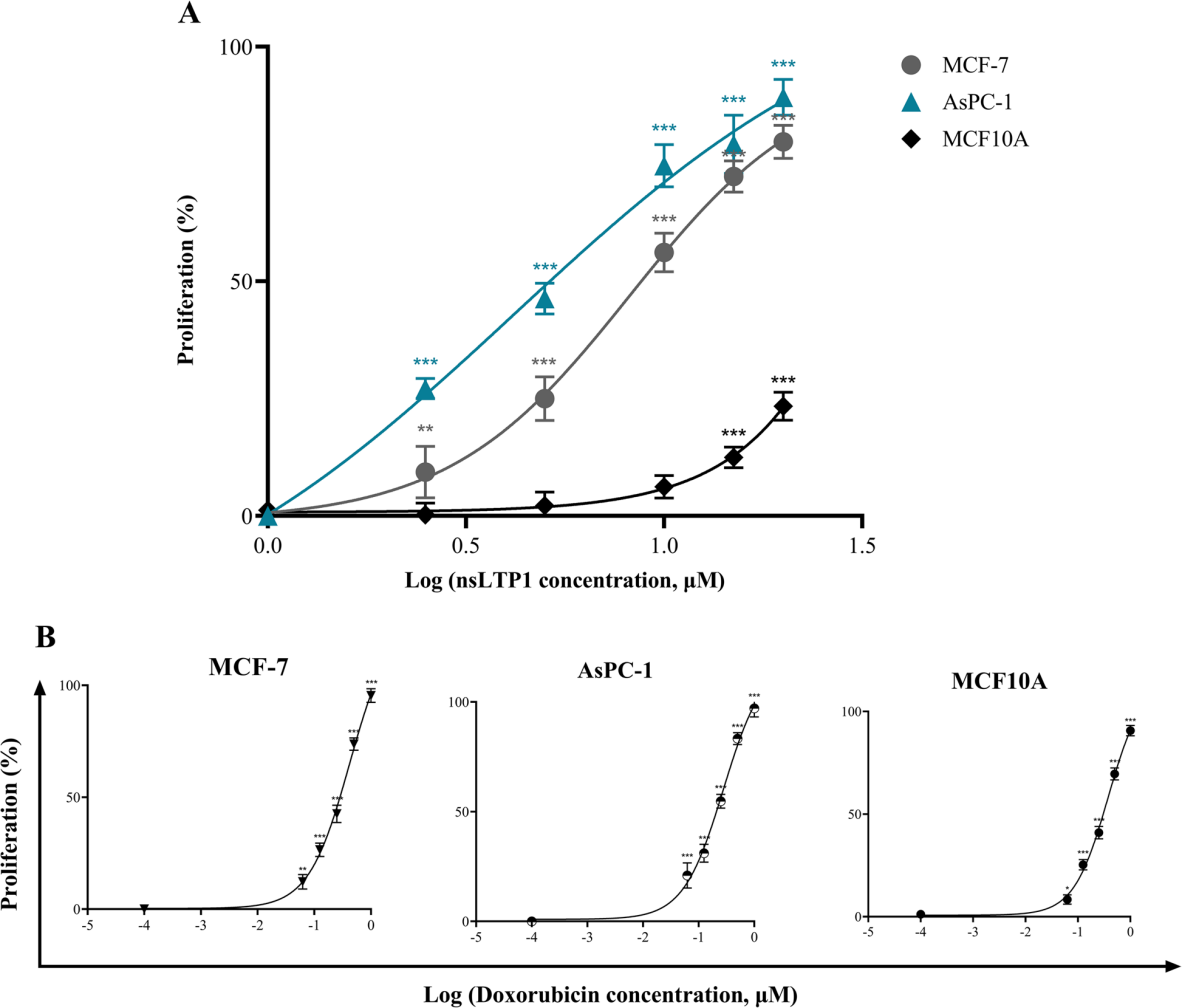
Dose-dependent cytotoxic activity of Ajwain nsLTP1 after 48 h treatment. Data from three independent experiments are presented with mean and standard deviation (SD) highlighted as *P < 0.05, **P < 0.01, ***P < 0.001. **A** nsLTP1 calculated IC_50_ values in MCF-7 and AsPC-1 were 8.21 μM and 4.17 μM, respectively. The pecentage of cells inhibition in MCF10A was 23% at 20 μM dose compared to the control. **B** Doxorubicin calculated IC_50_ values, 0.4224 ± 0.03 μM in MCF-7, 0.2786 ± 0.01 μM in AsPC-1, and 0.3767 ± 0.02 μM MCF10A

The activity of purified nsLTP1 from Ajwain seeds against normal breast cell line MCF10A and cancer cell lines MCF-7 and AsPC-1 were evaluated using the MTT assay. The nsLTP1 concentration in the sample was determined by Bradford assay and screened at different doses (2.5, 5, 10, 15, and 20 μM). In a dose-dependent manner, our results showed a potent cytotoxic effect in MCF-7 and AsPC-1 compared to the control cells (Fig. 1A). After 48 h treatment, the 20 μM dose significantly suppressed the survival of MCF-7 and AsPC-1 by 80% (***P < 0.001) and 89% (***P < 0.001), respectively. The IC_50_ concentrations were calculated using nonlinear regression analysis; for MCF-7, the value was 8.21 ± 0.79 μM, while for AsPC-1, 4.17 ± 0.34 μM, indicating that nsLTP1 shows a more cytotoxic effect against the pancreatic cancer cells compared to the breast cancer cells. However, the inhibition effect on MCF10A at a higher dose (20 μM) was estimated as 23%. Contrary to cancer cells, nsLTP1 did not exhibit the same degree of cytotoxicity against normal cells (Fig. 1A). Also, a dose-dependent inhibition was observed for doxorubicin (positive control) in all cell lines with IC_50_ values of 0.4224 ± 0.03 μM (MCF-7) 0.2786 ± 0.01 μM (AsPC-1), and 0.3767 ± 0.02 μM (MCF10A) as shown in Fig. 1B.

### Proapoptotic effects of nsLTP1

**Fig. 2:**
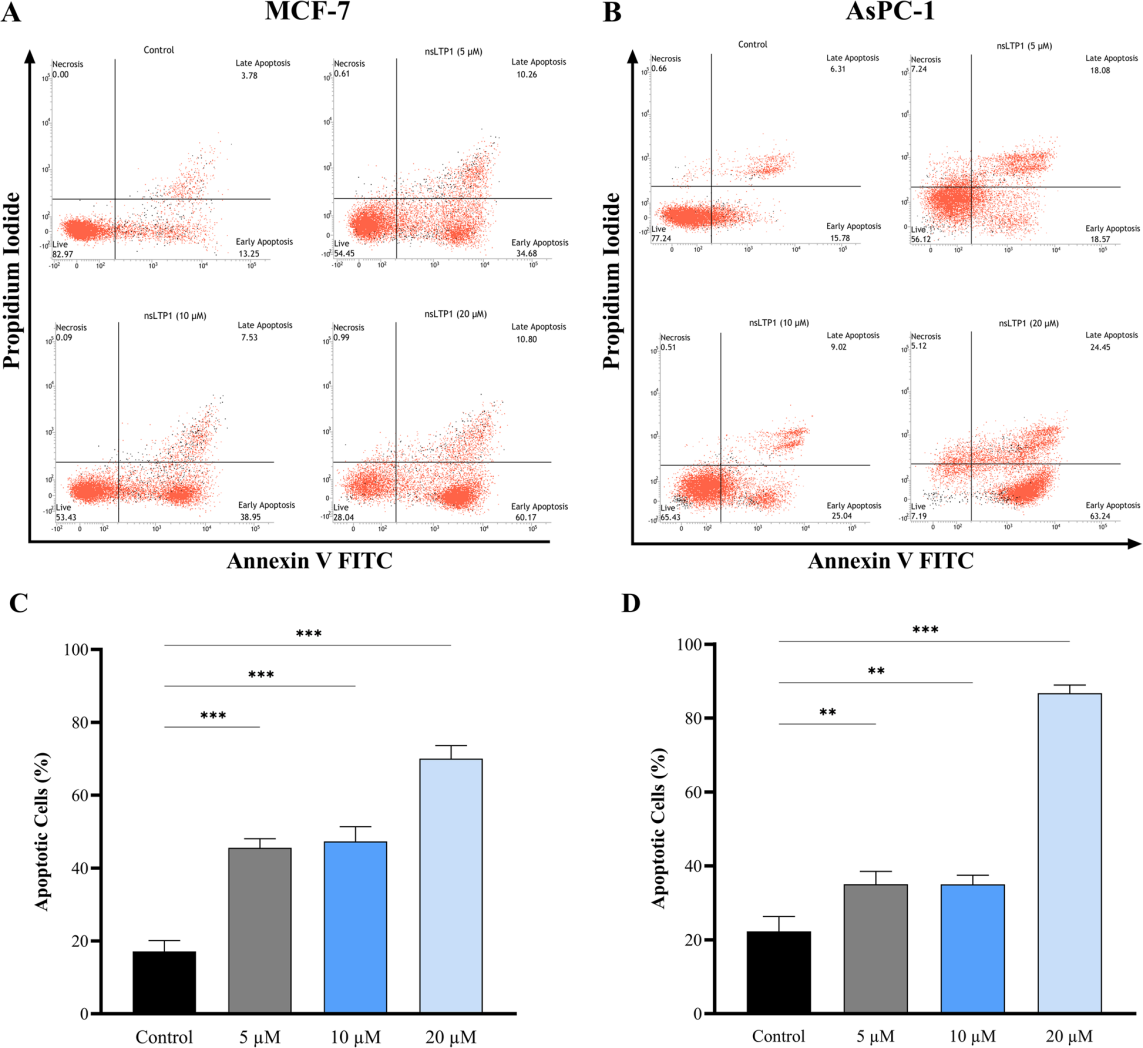
The apoptotic effect of Ajwain nsLTP1 using Annexin V-FITC/PI staining by flow cytometry. Live cells (Annexin V−, PI−), early apoptotic cells (Annexin V+, PI−), late apoptotic cells (Annexin V+, PI+), and necrotic cells (Annexin V−, PI+), (**A**) MCF-7 (**B**) AsPC-1 cells. The percentage of apoptosis among different concentration treatments is represented in MCF-7 cells (**C**) and AsPC-1 cells (**D**) compared to control cells. The sum of early apoptotic and late apoptotic cells was considered as the percentage of apoptosis. The data were presented as the mean ± SD of three independent experiments and significance are indicated by *P < 0.05, **P < 0.01, ***P < 0.001

To investigate the role of nsLTP1 in inducing apoptosis, MCF-7 and AsPC-1 cells were treated with various concentrations (5, 10, and 20 μM) and without nsLTP1 for 48 h. Apoptosis was detected using Annexin-V and Propidium Iodide (PI) staining, and the number of apoptotic cells was counted in the lower right (early apoptosis) and upper right (late apoptosis) quadrants. As depicted in Fig. 2A & B, the proportions of apoptotic cells in both cell lines were relatively increased depending on the concentration. In comparison with control, the apoptotic cells percentage at 20 μM was increased significantly in MCF-7 to 71% (***P < 0.001) (Fig. 2C) as well as in AsPC-1 to 88% (***P < 0.001) (Fig. 2D). At lower concentrations, a slight difference in the degree of apoptosis was observed in both cell lines, which may be due to differences in cancer cell types. For instance, in MCF-7 cells, there is more apoptosis observed (***P < 0.001) compared to AsPC-1 (**P < 0.01). Overall, we can safely conclude that nsLTP1 can induce apoptosis in multiple types of cancerous cells. The data were presented as the mean ± SD of three independent experiments.

### Effect of nsLTP1 on the expression of apoptosis-related genes

**Fig. 3:**
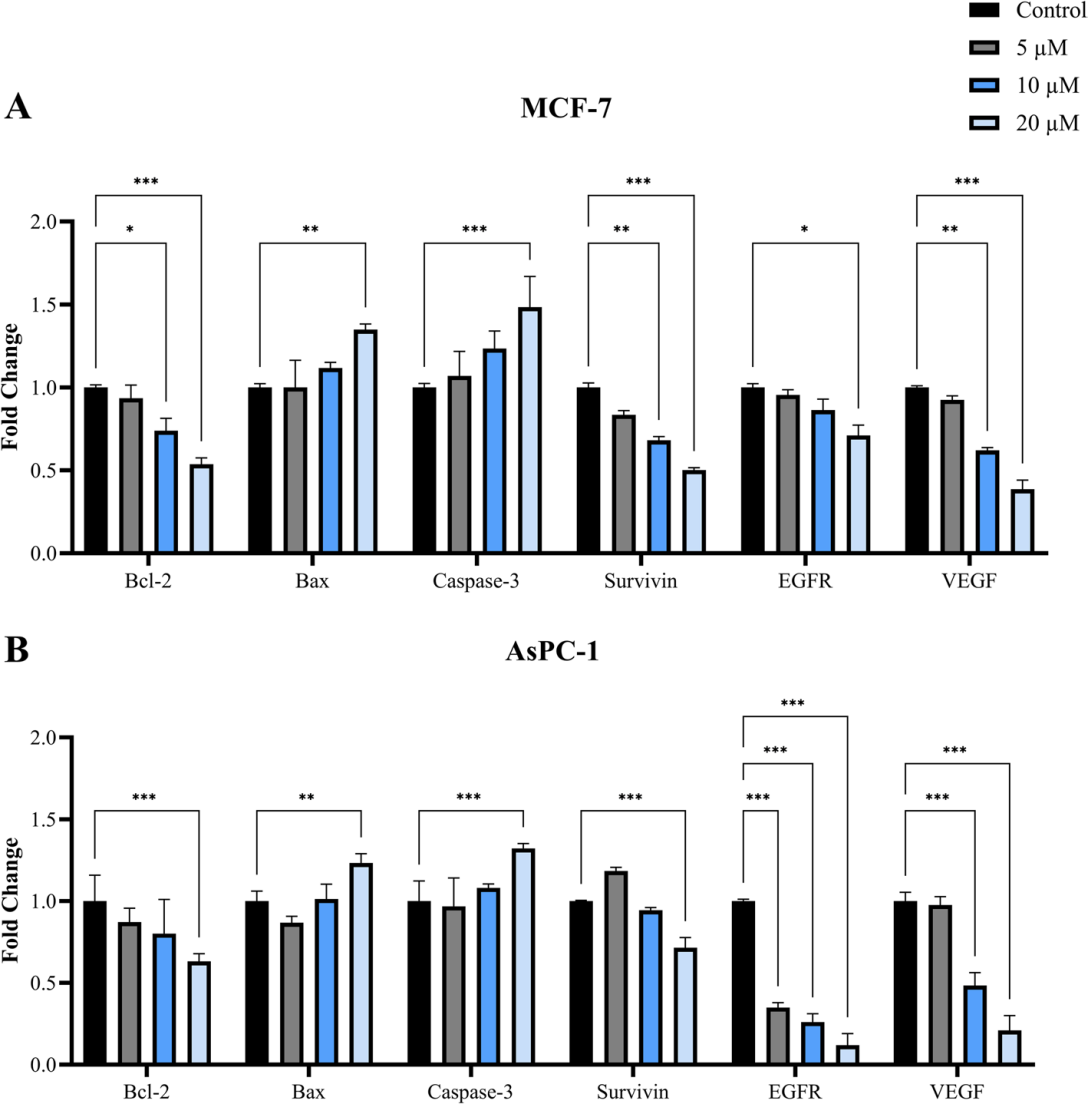
Ajwain nsLTP1 mechanism of action in inducing apoptosis in MCF-7 and AsPC-1. Both cell lines were treated with the same concentrations and compared to control cells. **A** In MCF-7, nsLTP1 promoted apoptosis by increasing the gene expression of proapoptotic proteins (Bax and Caspase-3) and decreasing the anti-apoptotic protein (Bcl-2). Also, it decreased the expression of other cancer-associated genes that promote cell survival, including, Survivin, EGFR, and VEGF. **B** In AsPC-1, nsLTP1 promoted apoptosis by upregulation of the gene expression of Bax and Caspase-3, and downregulation of Bcl-2, Survivin, EGFR, and VEGF. All data were obtained from three independent experiments and significance are indicated by *P < 0.05, **P < 0.01, ***P < 0.001

Further analysis was performed to explore the mechanism of nsLTP1 in inducing apoptosis in MCF-7 and AsPC-1. The gene expression of proapoptotic (Bax and Caspase-3) and cancer-associated genes which promote cell survival (Bcl-2, Survivin, EGFR, and VEGF) were analyzed in RT-PCR (Fig. 3). In MCF-7, a significant upregulation fold change was observed in Bax and Caspase-3 by 1.4-fold (**P < 0.01) and 1.5-fold (***P < 0.001), respectively, at 20 μM dose. Also, the same dose significantly caused downregulation in Bcl-2, Surviving, EGFR, and VEGF genes by 0.5, 0.5, 0.7, and 0.4 fold, respectively (Fig. 3A). In congruence with the MCF-7 findings, Bax in AsPC-1 cells was upregulated significantly by 1.2-fold (**P < 0.01); also, in Caspase-3, the fold change was noted at a significant level of 1.3-fold (***P < 0.001). On the other hand, we noted a significant downregulation in the fold change of Bcl-2, Surviving, EGFR, and VEGF genes by 0.6, 0.7, 0.1, and 0.2 fold, respectively (***P < 0.001), as shown in Fig. 3B. All the above data were compared to control cells.

### Effect of thermal treatments on nsLTP1 secondary structure

**Fig. 4:**
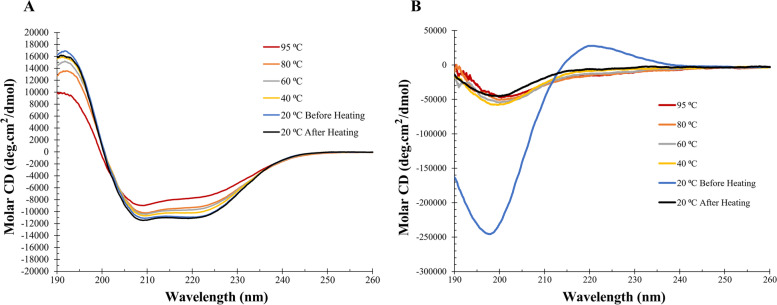
The thermal treatments effect on Ajwain nsLTP1 secondary structure. Collagen was used as a control in this experiment. **A** nsLTP1 CD spectra were collected at 20, 40, 60, 80, 95 °C, and after cooling down to 20 °C. There were minimal changes in the secondary structure at 95 °C; however, it is back to the original upon cooling it to 20 °C. **B** collagen was denatured as the temperature increased to 40 °C and remained denatured even after cooling it down to 20 °C

CD spectroscopy was used to investigate the stability of nsLTP1 at various temperatures. The spectra were recorded for nsLTP1 and the control (collagen) at temperatures ranging from 20 to 95 °C temperatures and after cooling to 20 °C, as shown in Fig. 4. The estimated secondary structure contents of the protein from Principal Component Regression (PCR) of the CD spectra indicated significant proportions of α-helical structure, as demonstrated in Table 1. The nsLTP1 showed the thermostable property. There were minimal changes in the secondary structure at 95 °C and after cooling down to 20 °C. The spectra before and after heating at 20 °C were almost similar. The positive maxima at 191 nm and double minima at 220 nm and 209 nm were the unique signatures of the protein (Fig. 4A). PCR analysis indicated a much lower content of β-sheet (21.9%) and turn (11.2%) compared to α-helix (32.4%) and other (34.5%) at 20 °C. Therefore, the CD spectrum of nsLTP1 implies a rich protein in α-helix. When heated to 95 °C, the maxima and minima decreased in intensity, reporting PCR analysis of β-sheet (2.5%), turn (15.5%), α-helix (43.1%), and other (38.9%). PCR analysis indicated an increase in α-helix and a corresponding decrease in β-sheet structure at 95 °C. After cooling down back to 20 °C, the spectrum almost matched the before heating, retaining native-like α-helix and β-sheet structures. In contrast, collagen was denatured as the temperature increased to 40 °C and remained denatured even after cooling it down to 20 °C (Fig. 4B). The data suggest that the Ajwain nsLTP1 is resistant to thermal denaturation, which only explains small conformational changes.

**Table 1:** Ajwain nsLTP1 secondary structure estimated by Principal Component Regression (PCR) analysis of CD spectra

Temperature	α-helix	β-sheet	Turn	Other
Before heating 20 °C	32.4%	21.9%	11.2%	34.5%
40 °C	35.5%	17.8%	12.0%	34.7%
60 °C	38.8%	13.5%	12.9%	34.8%
80 °C	43.1%	7.0%	14.0%	35.9%
95 °C	43.1%	2.5%	15.5%	38.9%
After heating 20 °C	34.9%	18.3%	11.6%	35.2%

### In vitro stability assay in human serum and medium containing FBS

**Fig. 5:**
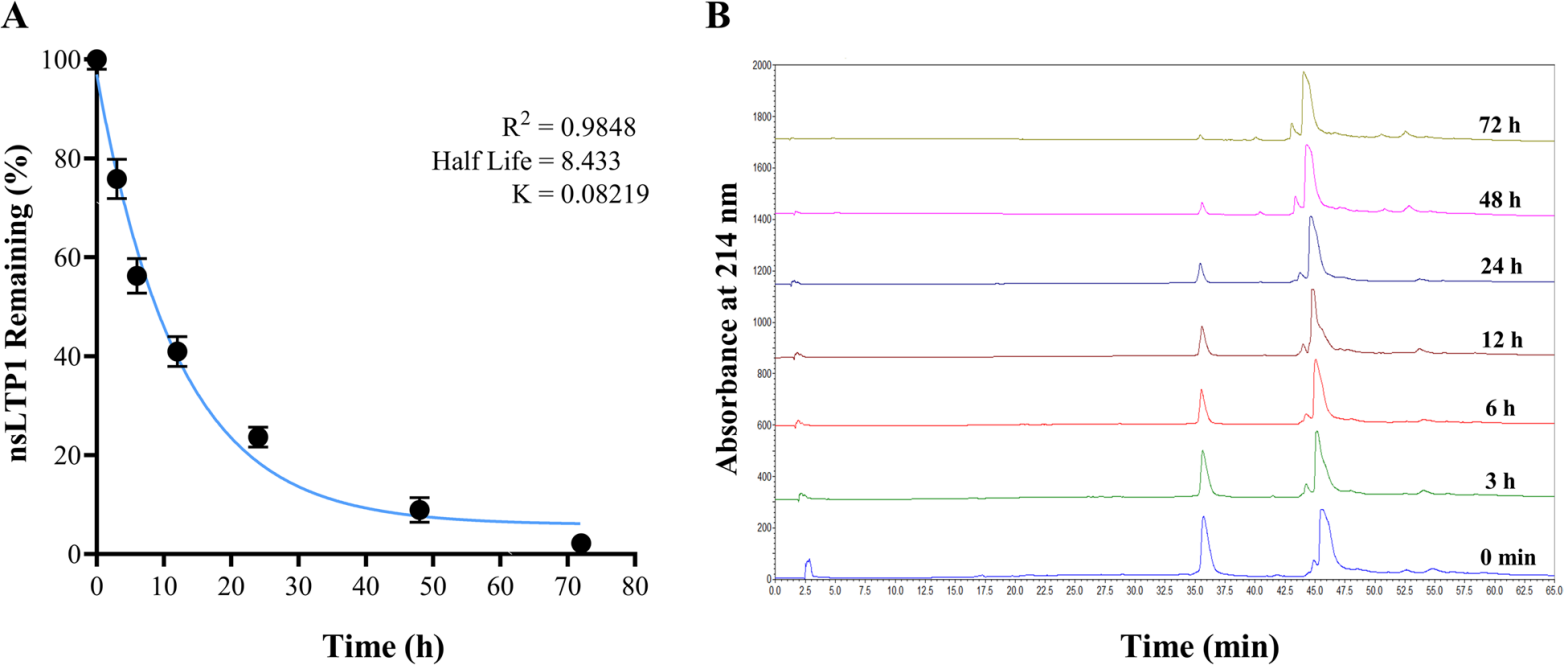
Stability of Ajwain nsLTP1 in human serum. **A** the percent remaining of nsLTP1 was quantified for specific incubation time points ranging from 0 to 72 h using the area under the curve (AUC) in analytical RP-HPLC. The trendline gives the half-life of ~ 8.4 h. Each point represents an average of three independent replicates. **B** Analytical RP-HPLC profile of nsLTP1 at retention time 36 min in human serum monitored at 214 nm

The stability of nsLTP1 was evaluated at 37 °C with a human serum to mimic the in vivo environment (Fig. 5) and with the cell culture medium containing 10% FBS (Supplementary Fig. S1). NsLTP1 showed high stability in human serum with a half-life of ~ 8.4 h at specific incubation time points ranging from 0 to 72 h, as illustrated in Fig. 5A. At 3 h incubation time, the remaining sample was 75% ± 4%, confirming the high stability. While, at 72 h, almost all the sample was degraded with a remaining portion of 2% ± 1%. The peak areas at a retention time of 36 min confirmed the slow degradation rate of nsLTP1 (Fig. 5B). Also, nsLTP1 was stable in the cell culture medium containing 10% FBS with an estimated half-life of 6.4 h (Supplementary Fig. S2). The remaining percentage of the sample at 3 h was 85% ± 5%. While, at 72 h, almost all the sample was degraded with a remaining portion of 4% ± 0.5%. The nsLTP1 and human serum were analyzed independently to confirm their elution retention times (Supplementary Fig. S3).

## Discussion

In 1975, Kader found the first plant nsLTP in potato tuber [39]. NsLTPs are small, highly soluble, and basic proteins found abundantly in all land plants. Their three-dimensional structure is stabilized by four disulfide bridges formed between an eight-cysteine motif (8-CM) in a typical sequence pattern C-Xn-C-Xn-CC-Xn-CXC-Xn-C-Xn-C [40]. Various hydrophobic molecules such as fatty acids, fatty acyl-coenzyme A, galactolipids, phospholipids, and phosphatidylglycerol can be transferred between plant membranes nonspecifically through nsLTPs. These proteins play a crucial role in many processes in plants, including stress resistance, defense against pathogen attack, reproduction, seed germination, and pollen adhesion [13]. However, nsLTPs biological activities are not limited to plants only; previous reports showed their ability to inhibit the growth of human tumor cells [23, 24]. Therefore, in this present study, we investigated the potential cytotoxic activity of nsLTP1 isolated from Ajwain seeds.

For a long period, Ajwain seeds small molecule extracts have been well known for their pharmacological properties; however, to the best of our knowledge, the macromolecule extracts (especially proteins) have not been studied for their potential cytotoxic activity against cancer cells. Thus, initially screened the crude proteins and gel filtration fractions against MCF-7 and AsPC-1; the preliminary data showed the maximum inhibition percentage occurred by the fractions, which contained the highest portion of nsLTP1. The purified nsLTP1 exhibited a cytotoxic effect against MCF-7 and AsPC-1 cells. The calculated IC_50_ values were 8.21 μM and 4.17 μM for MCF-7 and AsPC-1, respectively, in comparison to control cells. Moreover, these cytotoxic effects were compared to two nsLTPs previously isolated from *Foeniculum vulgare* (tested in MCF-7 with IC_50_ 6.98 μM) and *Brassica campestris* (tested in MCF-7 and Hep G2 with IC_50_ 1.6 μM and 5.8 μM, respectively) [23, 24]. Results indicate that nsLTPs from different plants act differently against various cancer cell lines; conversely, *Vigna radiata* nsLTP was inactive against MCF-7. Sequence alignment of these nsLTPs showed several differences in their amino acid sequences, which could be the reason for the variable activity observed.

The previous results were further supported by analyzing the number of apoptotic cells using the flow cytometric method, which showed consistent results with the cytotoxicity assay. It is suggested that inducing apoptosis might be an effective cancer treatment strategy [41]. Our results revealed that nsLTP1 possessed a significant ability to induce apoptosis in both MCF-7 and AsPC-1 cells. For MCF-7 cells, as compared to control cells, nsLTP1 induced apoptosis at concentrations of 5 and 10 μM, almost reaching 50%. Meanwhile, the highest apoptotic 71% was recorded at 20 μM. At 5 and 10 μM, nsLTP1 induced apoptosis in MCF-7 cells more than that observed in AsPC-1 cells. However, at 20 μM, the apoptotic percentage of nsLTP1 in AsPC-1 was higher than that found in MCF-7 cells compared to control cells, as shown in Fig. 2A & B.

The mechanism of apoptosis exerted by Ajwain nsLTP1 in MCF-7 and AsPC-1 was further evaluated based on the gene expression level. We analyzed several genes associated with tumor growth and its resistance to treatment, including Bcl-2, Bax, Caspase-3, Survivin, EGFR, and VEGF. The Bcl-2 protein family controls the mitochondrial apoptotic pathway through proapoptotic (Bax) and anti-apoptotic (Bcl-2) proteins. The Bax/Bcl-2 ratio imbalance will prevent the activation of executioner Caspase-3, which is essential in initiating downstream substrates cleavage involved in apoptotic changes [42]. This study observed a significant upregulation in Bax and Caspase-3 and downregulation in Bcl-2 after nsLTP1 treatment, indicating apoptosis activation in MCF-7 and AsPC-1. Survivin, a member of the inhibitor of apoptosis protein (IAP) family, inhibits caspases activation, which eventually leads to the inactivation of the apoptosis process. Survivin is highly expressed in cancer cells and fetal tissue; however, in terminally differentiated cells is undetected. Its effect on angiogenesis promotion is associated with its capability to promote vascular endothelial growth factor (VEGF) in cancer cells, which is considered a rate-limiting step in tumor growth [43]. In addition, epidermal growth factor receptor (EGFR) overexpression enhances Survivin expression in cancer cells through the phosphoinositide 3-kinases pathway [44]. Based on these findings, Survivin could potentially offer a new cancer therapeutic target distinguishing between transformed and normal cells [45]. The results showed that at the highest concentration (20 μM), nsLTP1 exhibited a significant upregulation of the expression levels of Bax and Caspase-3 genes in MCF-7 cells, compared to the control cells. Conversely, nsLTP1 possessed a downregulation effect towards the expression levels of Bcl-2, Surviving, EGFR, and VEGF genes. Regarding AsPC-1 cells, Bax and Caspase-3 genes expression levels were significantly upregulated by the action of nsLTP1; however, Bcl-2, Survivin, EGFR, and VEGF genes expression were downregulated in comparison to the control cells.

Protein-based drugs are becoming more important due to their ability to treat various diseases. Despite their unique characteristics, including high activity, strong specificity, and low toxicity, structural stability is a major hurdle for the pharmaceutical industries from fastly developing new therapeutic proteins. For instance, the high temperature causes conformational changes (unfold) in a protein structure, reducing their therapeutic effect or triggering severe immune responses [46]. Thus, we investigated the thermal stability of Ajwain nsLTP1 using CD spectroscopy. Interestingly, we found that nsLTP1 is a highly stable protein at 95 °C temperature, suggesting it has a melting temperature above 100 °C. In other studies, in situ heating of nsLTP1 from apple reveals that it unfolds slightly at elevated temperature and refolded upon cooling [47]., and no major changes were reported in the structure of LTP1 from barley in the temperature range of 20 °C to 90 °C [48]. These data are consistent with observations made regarding the thermostability of nsLTP1, possibly due to their unique presence of intramolecular disulfide bonds bestowing the characteristic of thermostability. In addition, after confirming the thermostability properties of nsLTP1, evaluated its stability in human serum. The estimated half-life was ~8.4 h which is considered close to the ideal half-life (12–48 h) [49]. Nevertheless, the promising results of Ajwain nsLTP1 in inhibiting the proliferation of breast and pancreatic cancers could be used for further investigations to prove its potential bioactive properties in an animal model.

## Conclusion

The Ajwain nsLTP1 protein exerts a potential cytotoxic activity against MCF-7 and AsPC-1 cancer cells and induces apoptosis via downregulation of anti-apoptotic proteins and upregulation of proapoptotic proteins. Also, nsLTP1 exhibited potent stability in high temperatures and in human serum, which may open an avenue towards the economic importance of plant protein-based drugs. However, to further confirm its cytotoxic effects, considering in vivo study is highly warranted.

## Supplementary Information

**Additional file 1: Supplementary Figure S1.** Stability of Ajwain nsLTP1 in cell culture medium containing 10% FBS. (A) nsLTP1 alone (B) cell culture medium containing 10% FBS alone (C-I) representing the percent remaining of nsLTP1 quantified for specific incubation time points ranging from 0 to 72 h using the area under the curve (AUC) in analytical RP-HPLC. **Supplementary Figure S2.** The percent remaining of nsLTP1 in the cell culture medium containing 10% FBS was quantified for specific incubation time points ranging from 0 to 72 h. The area under the curve (AUC) values reflect the nsLTP1 remaining percentage. The estimated half-life was ~ 6.4 h. Each point represents an average of three independent replicates. **Supplementary Figure S3.** Chromatographic profile of Ajwain (A) nsLTP1 alone eluted at 36 min (B) Human serum proteins eluted between 45 and 47 min. Samples were prepared in 100% cold methanol and analyzed by RP-HPLC. Column Aeris Widepore-C4 particle size 3.6 μm, pore size 200 Å (250 × 4.6 mm); gradient from 0 to 60% acetonitrile with 0.1% trifluoroacetic acid (TFA) in 65 min. The elution flow rate was 1 mL/min, and the absorbance was monitored at 214 nm.

## Data Availability

The datasets generated or analyzed during the current study are part of the S.O.A ongoing doctoral thesis but are available from the corresponding author upon reasonable request.

## Abbreviations

nsLTP: Nonspecific Lipid Transfer Protein
RT-PCR: Real-Time Polymerase Chain Reaction
PCR: Principal Component Regression
CD: Circular Dichroism
MCF-7: Human breast cancer
IC_50_: Half-maximal inhibitory concentration
AsPC-1: Human pancreatic cancer
Bcl-2: B-cell lymphoma 2
Bax: Bcl-2-associated X protein
IAP: Inhibitor of Apoptosis Protein
EGFR: Epidermal growth factor receptor
VEGF: Vascular endothelial growth factor
RP-HPLC: Reversed Phase-High Performance Liquid Chromatographic
TFA: Trifluoroacetic Acid
