# Supplementary material for: Cytotoxic activity of non-specific lipid transfer protein (nsLTP1) from Ajwain (Trachyspermum ammi) seeds
Source: BMC Complement Med Ther. 2022 May 16;22:135. doi: 10.1186/s12906-022-03616-y (PMC9112568; doi:10.1186/s12906-022-03616-y)

Supplementary information

Cytotoxic activity of non-specific lipid transfer protein (nsLTP1) from Ajwain *(Trachyspermum ammi)* seeds

Saud O. Alshammari[1,2], Taibah Aldakhil[1], Qamar A. Alshammari[1,3], David Salehi[1], Aftab Ahmed[1,*]

[1] Biomedical and Pharmaceutical Sciences, Chapman University School of Pharmacy, Irvine, CA 92618, USA

[2] Department of Plant Chemistry and Natural Products, Faculty of Pharmacy, Northern Border University, KSA

[3] Department of Pharmacology and Toxicology, Faculty of Pharmacy, Northern Border University, KSA

[4] Department of Pharmaceutical Chemistry, College of Pharmacy, Prince Sattam Bin Abdulaziz University, Al-Kharj, KSA

*Corresponding author

Aftab Ahmed
Chapman University School of Pharmacy
9401 Jeronimo Road
Irvine, CA 92618
USA
Tel: (714) 516-5465
Fax: (714) 516-5481
aahmed@chapman.edu

Supplementary Figure S1: Stability of Ajwain nsLTP1 in cell culture medium containing 10% FBS. (**A**) nsLTP1 alone (**B**) cell culture medium containing 10% FBS alone (**C-I**) representing the percent remaining of nsLTP1 quantified for specific incubation time points ranging from 0 to 72 h using the area under the curve (AUC) in analytical RP-HPLC.

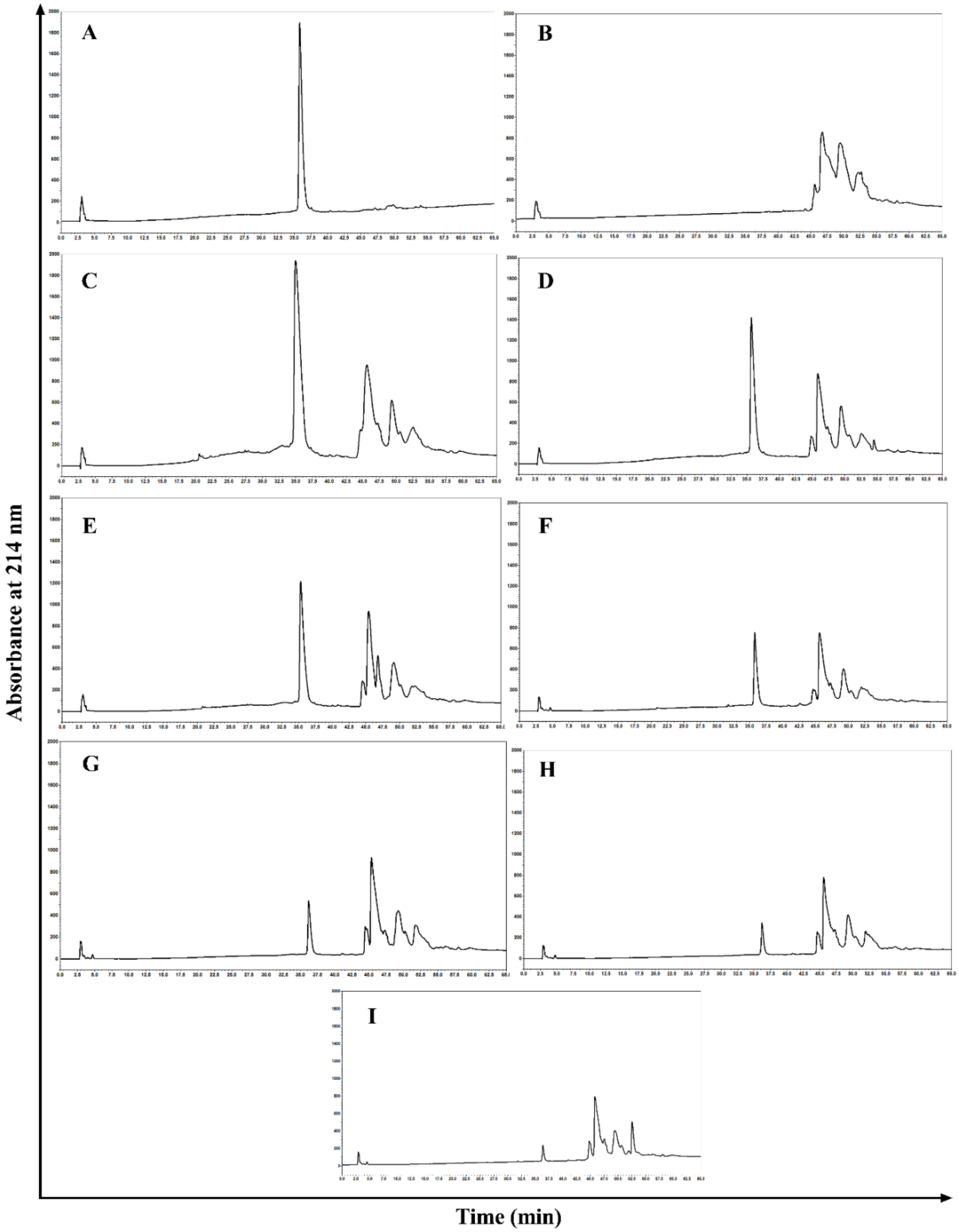

Supplementary Figure S2: The percent remaining of nsLTP1 in the cell culture medium containing 10% FBS was quantified for specific incubation time points ranging from 0 to 72 h. The area under the curve (AUC) values reflect the nsLTP1 remaining percentage. The estimated half-life was ~ 6.4 h. Each point represents an average of three independent replicates.

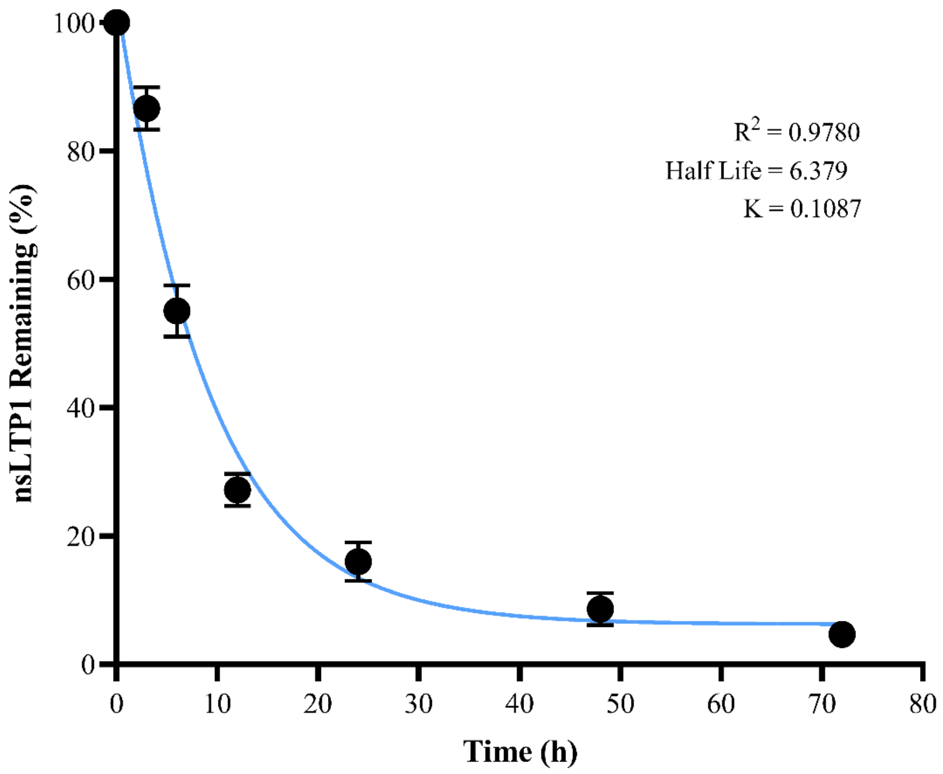

Supplementary Figure S3: Chromatographic profile of Ajwain (A) nsLTP1 alone eluted at 36 min (B) Human serum proteins eluted between 45-47 min. Samples were prepared in 100% cold methanol and analyzed by RP-HPLC. Column Aeris Widepore-C4 particle size 3.6 µm, pore size 200Å (250 × 4.6 mm); gradient from 0–60% acetonitrile with 0.1% trifluoroacetic acid (TFA) in 65 min. The elution flow rate was 1 mL/min, and the absorbance was monitored at 214 nm.

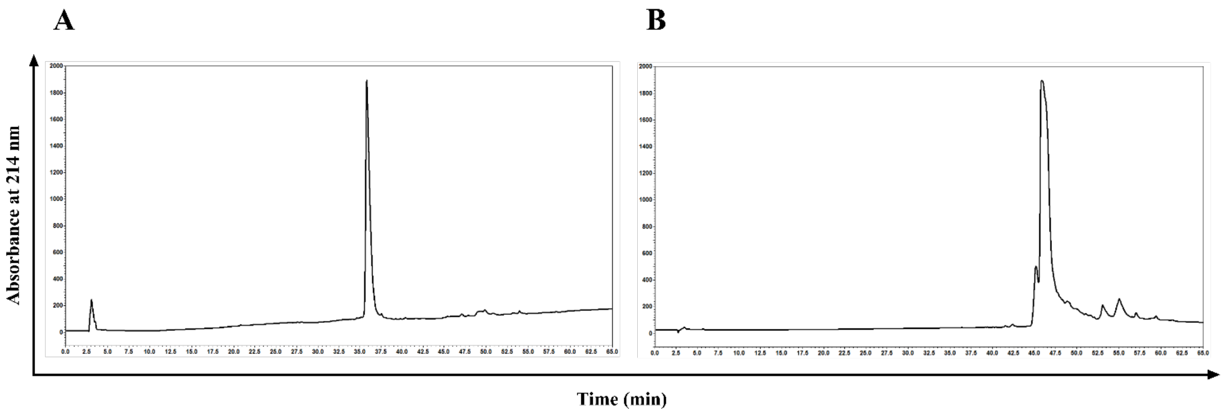

Supplement: Supplementary file 1 — Additional file 1: Supplementary Figure S1. Stability of Ajwain nsLTP1 in cell culture medium containing 10% FBS. (A) nsLTP1 alone (B) cell culture medium containing 10% FBS alone (C-I) representing the percent remaining of nsLTP1 quantified for specific incubation time points ranging from 0 to 72 h using the area under the curve (AUC) in analytical RP-HPLC. Supplementary Figure S2. The percent remaining of nsLTP1 in the cell culture medium containing 10% FBS was quantified for specific incubation time points ranging from 0 to 72 h. The area under the curve (AUC) values reflect the nsLTP1 remaining percentage. The estimated half-life was ~ 6.4 h. Each point represents an average of three independent replicates. Supplementary Figure S3. Chromatographic profile of Ajwain (A) nsLTP1 alone eluted at 36 min (B) Human serum proteins eluted between 45 and 47 min. Samples were prepared in 100% cold methanol and analyzed by RP-HPLC. Column Aeris Widepore-C4 particle size 3.6 μm, pore size 200 Å (250 × 4.6 mm); gradient from 0 to 60% acetonitrile with 0.1% trifluoroacetic acid (TFA) in 65 min. The elution flow rate was 1 mL/min, and the absorbance was monitored at 214 nm. [file 12906_2022_3616_MOESM1_ESM.pdf]
